# From first contact to challenge: Lung immune modulation by single-sex *Schistosoma mansoni* infections

**DOI:** 10.1371/journal.pntd.0014814

**Published:** 2026-10-27

**Authors:** Miriam Bischofsberger, Tim A. Dannenhaus, Sanja U. Schönberger, Emil C. Reisinger, Micha Löbermann, Martina Sombetzki

**Affiliations:** Department of Internal Medicine, Division of Infectious Diseases, Nephrology, Endocrinology and Tropical Medicine, Rostock University Medical Center, Rostock, Germany; Cyprus International University: Uluslararasi Kibris Universitesi, CYPRUS

## Abstract

**Background:**

Schistosomiasis is a chronic parasitic disease caused by blood flukes of the genus *Schistosoma* and remains a major public health concern in endemic regions. Following skin penetration, schistosomula migrate through multiple host tissues before maturing into adult worm within the mesenteric vasculature. Immunological priming critically influences host responses during secondary infections, yet the importance of prior single-sex *Schistosoma mansoni* infection on pulmonary recall immunity remains largely unknown. Since the lungs serve as an important immunological checkpoint during schistosomula migration, we herein investigated how single-sex and bisexual priming influence the pulmonary immune landscape upon reinfection.

**Methodology:**

Using a murine reinfection model, mice were infected with male, female or mixed-sex *S. mansoni* cercariae and subsequently challenged with a bisexual infection. Pulmonary immune responses were analyzed by expression of key-regulatory genes, high-dimensional flow cytometry, tSNE/FlowSOM clustering and immune cell phenotyping.

**Conclusions:**

Single-sex priming resulted in distinct immune responses compared to bisexual infection. Male priming was associated with reduced pulmonary worm burden and a preferential enrichment of circulating CD4^+^ central and effector memory T cells, whereas female priming promoted the accumulation of CD4^+^ resident memory T cells together with broader alterations in pulmonary myeloid cell populations. Our findings demonstrate that the sex of primary *Schistosoma mansoni* infection differently programs pulmonary immune recall responses, particularly through distinct organization of the CD4^+^ memory T cell compartment. The association between male priming, enrichment of circulating memory T cell subsets and reduced pulmonary worm burden identifies pulmonary memory T cell organization as a potential correlate of protection during reinfection.

## Author summary

The present study demonstrated that single-sex *Schistosoma mansoni* priming profoundly shapes the pulmonary immune response upon subsequent bisexual challenge infection. Building upon previous observations that single-sex infections differently affect hepatic pathology, we investigated whether these priming effects are already detectable during the critical pulmonary migration phase of reinfection.

Our results show that the sex of the primary infection establishes distinct pulmonary immune recall programs. Male priming was associated with reduced pulmonary worm burden and a preferential enrichment of circulating CD4^+^ central and effector memory T cells, whereas female priming promoted enrichment of CD4^+^ resident memory T cells together with broader alterations of pulmonary myeloid cell populations. In contrast, bisexual priming generated a different immune profile likely shaped by continuous exposure to both worm and egg antigens. These findings identify pulmonary memory T cell organization as a potential mechanism contributing to the different outcomes of reinfection following male and female single-sex infections.

## Introduction

Schistosomiasis remains one of the most prevalent and devastating neglected tropical diseases worldwide, imposing a substantial burden on global health and socioeconomic development. The disease, caused by trematode blood flukes of the genus *Schistosoma*, affects more than 200 million people worldwide, with over 700 million at risk of infection—predominantly in sub-Saharan Africa, the Middle East, and South America. Recent reports of autochthonous cases in southern Europe underscore the potential for transmission beyond traditionally endemic regions, driven by climate change and human migration [1,2].

The pathogenesis of schistosomiasis has been attributed to the host immune response against parasite eggs that become trapped within blood vessels and surrounding tissues, resulting in granulomatous inflammation and fibrosis [3,4]. However, studies using single-sex infections have revealed that, in addition to parasite eggs, adult worms are also capable of modulating host immunity. In our long-term single-sex infection model, infection of mice with either male or female worms for 52 weeks induced persistent, organ-damaging, non-polarized T cell-mediated inflammatory responses [5]. Consistent with these findings, Boissier et al. reported that male worms elicit a more pronounced immune activation than females [6], whereas female *Schistosoma mansoni* exhibit greater resistance to Praziquantel and employ distinct immune evasion strategies to modulate the host’s immune response [7]. In line with this, transcriptomic analyses of spleens from single- and bisexually infected mice demonstrated distinct gene expression profiles depending on infection type [8]. The recent establishment of controlled human infection models using either male or female *Schistosoma mansoni* cercariae has enabled investigation of egg-free schistosomiasis in humans and confirmed that adult worms alone induce distinct immune responses [9–11]. These observations indicate that the adult worms play an active role in regulating the host immune response, independently of the parasite eggs.

To date, praziquantel is the only available treatment for schistosomiasis. However, praziquantel only affects adult worms and has no effect on other stages of the parasite’s life cycle. Experimental vaccination with radio-attenuated (RA) cercariae demonstrated that protective immunity can reduce challenge worm burden by up to 80% and is primarily established during the pulmonary migratory phase, identifying the lung as a key site of immune-mediated parasite elimination [12–14]. Protective immunity to schistosomiasis in mice has been associated with type-1 immune responses, whereas in humans Th2 responses seem to be the key to resistance. Several studies indicate that the parasite-specific IgE, IgE receptor-expressing eosinophils and B-cells, as well as cytokines such as IL-5 and IL-4, contribute to resistance against reinfection in humans [15–21]. T cell memory subsets have gained increasing attention because they provide rapid and highly effective protection against previously encountered pathogens. Central memory T cells (T_CM_) and effector memory T cells (T_EM_) continuously recirculate through the blood and secondary lymphoid organs or between peripheral tissues and the bloodstream, enabling local responses upon antigen re-encounter. In contrast, tissue-resident memory T cells (T_RM_) permanently reside within non-lymphoid tissues, particularly at epithelial barrier sites such as the skin, respiratory, gastrointestinal and reproductive tracts, where they provide rapid local immune protection independently of circulating T cells [22–25].

While infection-associated liver and intestinal damage is considered an important feature of infection with *S. mansoni,* the lung is a crucial but often overlooked organ throughout the entire life cycle. In the early phase of infection, schistosomula migrate through the pulmonary vascular system between days 4–20 post infection, where they encounter and interact with local immune cell populations, leading to local inflammation and triggering a Th2-biased response [26–28]. During this phase, the predominant pulmonary symptoms are cough and lesions of the lung tissue caused by immune cell infiltrates, resulting from a systemic hypersensitivity response to lung-stage larvae [29,30]. However, the exact causes of pulmonary pathology during lung migration have not yet been thoroughly investigated [31].

The migration through the lung, whether via lung tissue or the vascular system, is an essential part of many parasitic worm lifecycles, thus passing the lung is not only disadvantageous. The pulmonary environment provides favorable conditions for larval development while simultaneously exposing parasites to mechanical constraints and host immune defenses [32,33]. Migration time of *S. mansoni* through the lung is between 30–35 hours in comparison to 6–16 hours in systemic and intestinal capillary beds suggesting the lung as a great migratory obstacle [34]. Lung migration confronts migrating larvae with several challenges, including elongation through narrow capillaries and the associated risk of rupturing into alveoli. Overcoming these challenges requires a wide range of immune evasion strategies, such as coating in host antibodies and evading complement-mediated killing. Mainly the prolonged transit through the lungs makes this stage a possible target of elimination [35–37].

We recently demonstrated that this very early stage (day 4 and 16 post infection) is characterized by a unique immune cell landscape that differs markedly between single-sex and bisexual infections [38]. Furthermore, in previous studies, we pointed out that infections with a single sex can influence both worm burden and pathology in a subsequent infection. Primary infections of mice with male worms reduced worm and egg burden in infections in the liver [39], whereas infections with female worms were associated with reduced liver fibrosis, smaller granulomas, and a dampened Th2-type immune response [40].

In this study, we aimed to address the existing knowledge gap regarding how single-sex priming affects the pulmonary phase of challenge infection, thereby contributing to a better understanding of early *S. mansoni* infection. Understanding how such pre-existing immune environments influence subsequent challenge infections may provide key insights into the balance between protective and pathological immunity in schistosomiasis.

## Materials and methods

### Ethics

All experiments involving the infection of mice were conducted in strict adherence to the regulatory guidelines set forth by the German Society for Laboratory Animal Science and the European Health Law of the Federation of Laboratory Animal Science Associations. The animal experimentation protocols underwent rigorous review and received approval from the Mecklenburg-Vorpommern State Office for Agriculture, Food Safety, and Fisheries (TVA 7221.3-1-70/21–1). Stringent measures were implemented to mitigate and minimize any potential suffering experienced by the animals.

### *Schistosoma mansoni* infection of mice

The life cycle of *Schistosoma mansoni,* Belo Horizonte strain, was maintained by infection of *Biomphalaria* (*B.*) *glabrata* freshwater snails as intermediate hosts and 6–8-week-old NMRI mice as definitive hosts. Infection of the snails was carried out in two ways. In order to obtain only male or female cercariae for subsequent infection of the mice, *B. glabrata* snails were exposed to a single *S. mansoni* miracidium. This resulted in a single-sex infected snail, as the sex of *S. mansoni* is already determined in the eggs [41,42]. In mixed-sex infections, *B. glabrata* snails were exposed to a miracidia suspension obtained from an infected mouse liver. After six weeks of infection, individual snails were subjected to a light stimulus (day light lamp, 10,000 Lux) for about 2 hours to induce the shedding of cercariae. In the case of single-sex infected snails, the sex determination of cercariae was conducted by real-time quantitative polymerase chain reaction (qPCR) using primers specific for the female sex. Briefly, mice were individually placed in beakers containing 45–50 mL of ankle-deep aquarium water with a predetermined number of cercariae for 2 h. To analyze the inflammatory environment in the lung during challenge infection, C57BL/6j mice were infected with 100 cercariae male-only, female-only or male and female for six weeks and then reinfected with 100 mixed-sex cercariae (n = 7, respectively) for six days. Naive mice (n = 3) and mice infected with 100 mixed-sex cercariae for only six days (n = 7) served as control groups (Fig 1).

**Fig 1:**
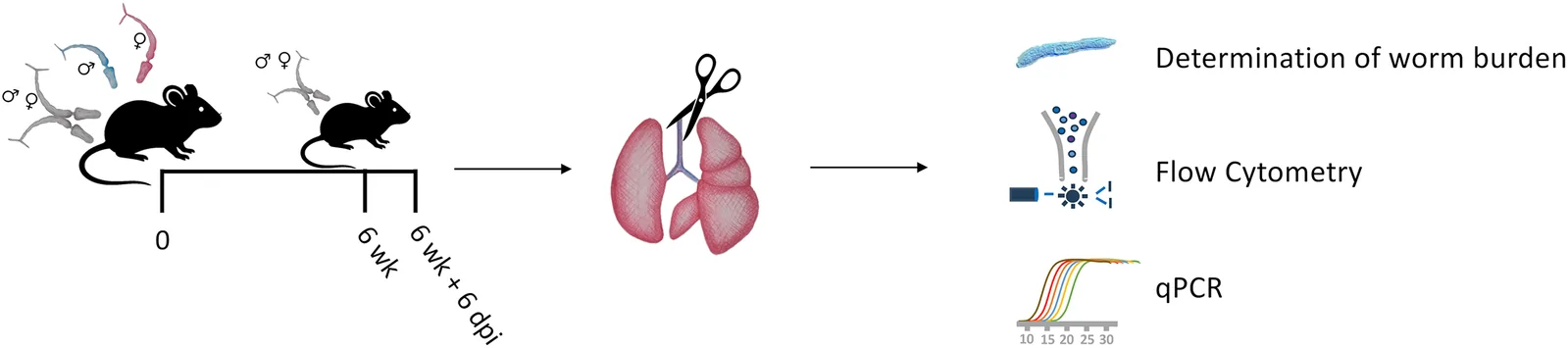
Experimental design. Mice were first infected with male, female or both *Schistosoma mansoni* cercariae over 6 weeks and then bisexually reinfected for 6 days. The worm burden, the cellular immune environment (using flow cytometry), and gene expression (using quantitative real-time-PCR) were analyzed in the lung tissue. wk: week; dpi: days post infection.

The mice were euthanized by final blood collection under ketamine/xylazine anesthesia. After blood collection the lungs were collected for determination of the worm burden (left lung) and the immune environment in the lung upon challenge infection.

### Determination of worm burden

The left lung lobe was minced with two sharp tweezers in a petri dish. The minced lungs were incubated in medium (RPMI 1640 containing 100 U/mL penicillin, 100 µg/mL streptomycin and 10% FBS). The emerged lung-stage larvae were counted microscopically after 24 h of incubation at 37 °C and 5% CO_2_.

### Quantitative real-time PCR analysis

Total RNA was extracted from snap frozen lung tissue (middle lobe) using the RNeasy Plus Mini Kit (Qiagen, Germany) and reversely transcribed into cDNA using a High-capacity cDNA Reverse Transcriptase Kit (Thermo Fisher, USA) according to the manufacturer’s instructions. All samples were analyzed in technical triplicates with a standard deviation of <0.25. RT-PCR was performed using the following TaqMan Gene Expression Assays: *tnf-α* Mm00443258_m1, *icam-1* Mm00516023_m1, *ifn-γ* Mm01168134_m1, *il-13* Mm00434204_m1, *foxp3* Mm00475162_m1, *hmox* Mm00516005_m1, *il-1β* Mm00434228_m1, *il-5* Mm00439646_m1, *il-10* Mm01288386_m1, *retnla* Mm00445109_m1, *il-33* Mm00505403_m1 and *nos-2* Mm00440502_m1. Gene expression was normalized to the endogenous reference *Gapdh* (Rodent GAPDH control reagent, Thermo Fisher, USA) and presented as relative expression levels normalized to the naive control group.

### Flow cytometric analysis

Single-cell suspensions from lung tissue were prepared using a defined segment in the lung (superior, inferior and post-caval lobe) that had been digested beforehand. The tissue was minced and incubated in 3 ml RPMI medium (RPMI 1640 with 100 U/ml penicillin and 100 µg/ml streptomycin) supplemented with 0.26 U/ml Liberase and 166 U/ml DNase I for 45 min at 37 °C. The digested tissue was then passed through a cell strainer and washed with cold PBS, followed by lysis of erythrocytes with ACK lysis buffer (Thermo Fisher, USA). Cell counts were determined using the CASY TT cell counter (OLS-Omni Life Science, Germany). A total of 10^6^ cells/sample were stained with Zombie NIR Fixable Viability Kit (BioLegend) for 20 min in PBS followed by a 20 min of incubation with TruStain FcX PLUS (anti-mouse CD16/32) (clone: S17011E) and True-Stain Monocyte Blocker (BioLegend). Blocked cells were then stained with appropriate fluorochrome-conjugated antibodies: anti-CD45-APC-Fire810 (clone: 30-F11), anti-CD64-PercP-eFluor710 (clone: X54-5/7.1), anti-CD11c-AlexaFluor647 (clone: N418), anti-CD11b-BrilliantViolet785 (clone: M1/70), anti-CD19-Super Bright600 (clone: eBio1D3), anti-Ly6G-BrilliantViolet650 (clone: 1A8), anti-SiglecF-BB515 (clone: E50-2440 RUO), anti-CD3-BrilliantViolet510 (clone: 17A2), anti-CD4-PerCP-Cy5.5 (clone: GK1.5), anti-CD8-BrilliantViolet711 (clone: 53-6.7), anti-CD62L-VioBlue (clone: MEL-H2.100), anti-CD44-VioBright R720 (clone: REA664), anti-CD69-PE-Cyanine 5 (clone: H1.2F3), and anti-CD103-PE-Fire810 (clone: QA17A24). The cells were fixed and permeabilized using the Transcription Factor Staining Buffer Set (Miltenyi Biotec, Germany) and prior to staining of the intracellular antibodies, blocked again with TruStain FcX PLUS (anti-mouse CD16/32) (clone: S17011E) and True-Stain Monocyte Blocker (BioLegend). Afterwards cells were stained with intracellular antibodies: anti-RORγt-APC (clone: REA278), anti-PU.1-AlexaFluor488 (clone: 7C2C34), anti-GATA3-PE/Vio615 (clone: REA174), anti-T-bet-PE-Cy7 (clone: 4B10), anti-FoxP3-BrilliantViolet421 (clone: MF-14), anti-CD3-BrilliantViolet510 (clone: 17A2) and anti-IL-22-PE (clone: Poly5164). Cytometric analyses were performed using the Cytek Aurora (Cytek Biosciences, USA) and data were processed using FlowJo Software (v10.8.1, Tree Star Inc. CA, USA) (for a representative gating strategy see S1 Fig.). For high-dimensional analysis, live CD45^+^ cells were exported from the gated flow cytometry data and equal numbers of events per sample were concatenated prior to dimensionality reduction. tSNE analysis was performed in FlowJo using the indicated fluorescence parameters to visualize the cellular landscape of pulmonary immune cells. FlowSOM clustering was subsequently applied to the concatenated CD45^+^ dataset to identify phenotypically distinct cell clusters based on marker expression. A total of 18 FlowSOM clusters were generated and manually annotated according their expression profiles of lineage- and activation-associated markers (S2 Fig.). Cluster annotations were used to compare distribution patterns between experimental groups and to visualize population-specific marker expression. For T cell-focused visualization CD3^+^ cells were exported and equal numbers of events per sample were concatenated prior to tSNE analysis.

**Statistics:** Statistical analysis was performed using GraphPad Prism 10 (GraphPad Software, USA). After testing the data for normality using the Shapiro-Wilk test, normally distributed data were tested for homogeneous variances using Levene’s test and analyzed with an ordinary one-way ANOVA, followed by a Dunnett’s test. Data sets that failed normality tests were compared using the Kruskal–Wallis test followed by a Dunn’s post-hoc test. Values are expressed as means or medians, respectively. For all statistical analyses, *p* values < 0.05 were considered significant. ^* #^ *p* < 0.05, ^** ##^ *p* < 0.01, ^*** ###^ *p* < 0.001. The Heatmap was generated in R (version 4.5.2) using z-score normalized mean fluorescence intensity (MFI) values exported from FlowJo. MFI values were averaged within each experimental group for each marker. Group mean MFIs were subsequently standardized separately for each marker by calculating z-scores across the four experimental groups using the scale() function in R. The resulting z-scores therefore represent the relative MFI oof each group compared with the mean MFI across all groups for the respective marker. The heatmap was then generated using the pheatmap package without additional scaling.

## Results

### Priming of mice influences the worm burden of challenge infections during lung migration

Mice were infected with 100 male, female or mixed-sex cercariae for 6 weeks and were then challenged with mixed-sex cercariae for 6 days. Lung-stage larvae out of the left lobe were counted to determine the worm burden of challenge infection. As control served mice only infected for 6 days with mixed-sex cercariae. Female pre-infection resulted in the highest challenge worm burden, which was significantly increased compared with male and bisexual pre-infection, whereas the lowest worm counts were observed in the bisexually pre-infected group (Fig 2).

**Fig 2:**
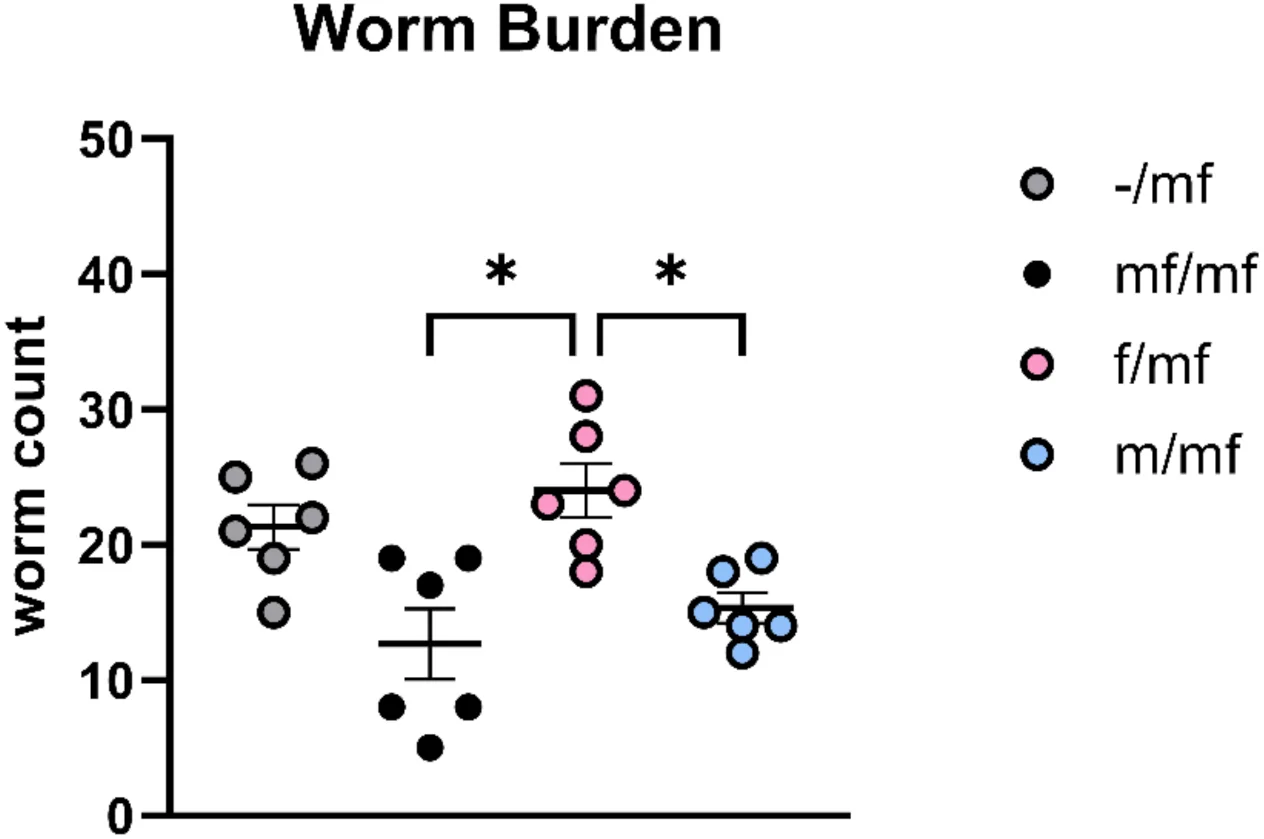
Infection with male *Schistosoma mansoni* cercariae results in a significantly lower worm burden of challenge infection in the lung compared to female infection. Worm burden assessed in the left lung lobe of mice (n = 6) infected with *Schistosoma mansoni* for 6 weeks with female, male or mixed-sex and reinfected bisexually for 6 days. As baseline, mice were only infected with mixed-sex cercariae for 6 days. m: male; f: female; mf: male and female. Data are presented with mean with SEM. *P* values < 0.05 were considered statistically significant. * *p* < 0.05.

### Single-sex priming leads to a non-polarized Th1/Th2 response at the gene level with a shift towards regulatory T cell response

To characterize the immune response during lung migration of challenge infection we performed quantitative real-time PCR on key regulating genes. Fig 3A shows relative gene expression with genes associated to Th1, Th2 and regulatory T (Treg) cell responses.

**Fig 3:**
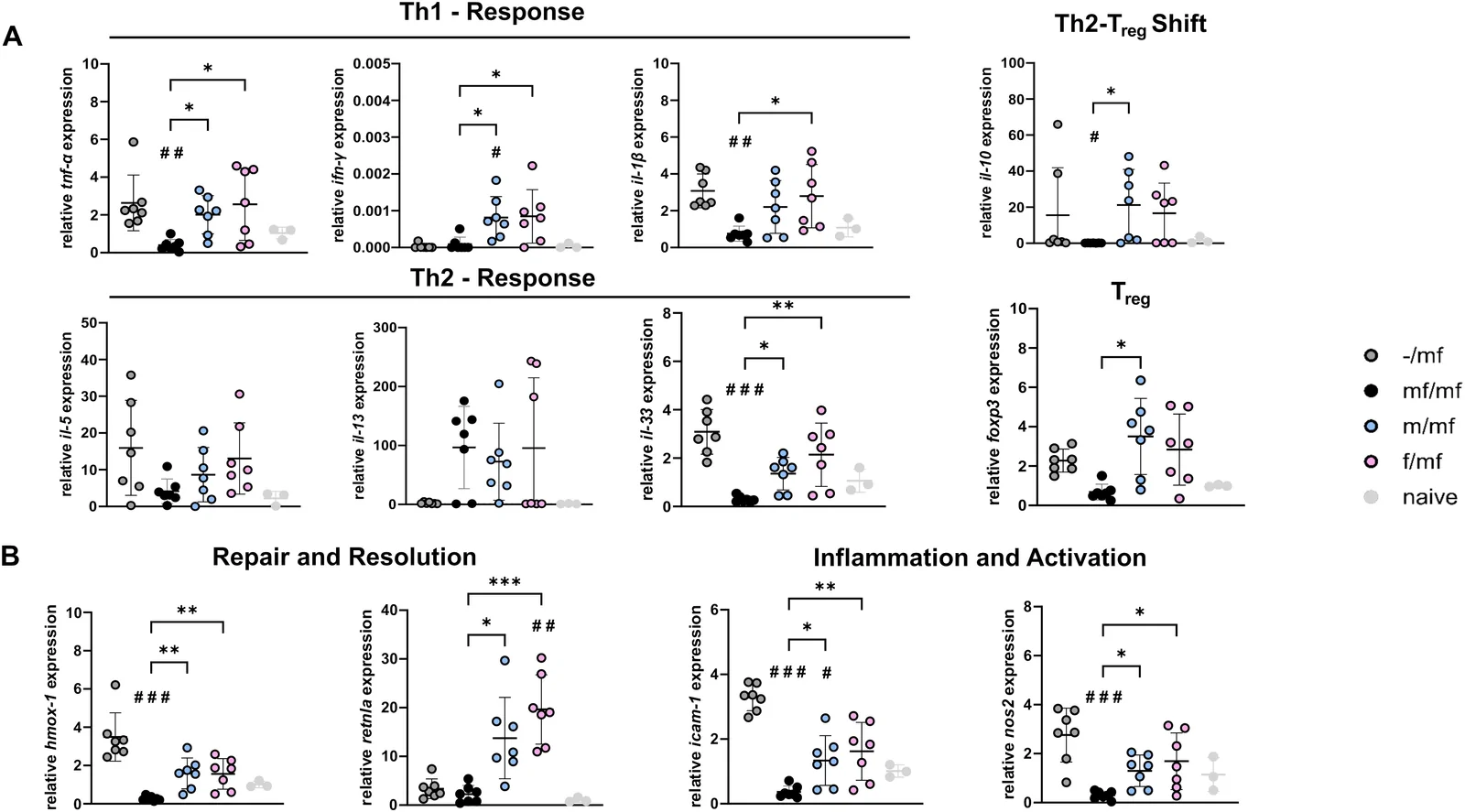
Single-sex priming with *Schistosoma mansoni* cause stronger expression of inflammation associated genes in the lung of challenge infected mice than bisexual infection. Relative gene expression was analyzed in the lungs of mice infected with male, female or both *Schistosoma mansoni* cercariae for 6 weeks and challenged bisexually for 6 days by real-time qPCR. Naive and non-primed bisexually infected mice served as control. **(A)** Th1 associated genes (*tnf-α*, *ifn-γ*, *il-1β*), Th2 associated genes (*il5*, *il-13*, *il-33*), shift Th2 to regulatory T cells (T_reg_) (*il-10*) and T cell associated *foxp3*. **(B)** Repair and Resolution related *hmox-1* and *retnla* and *icam-1* and *nos2* as inflammatory and activation parameters. M: male; f: female; mf: male and female; -: no infection. Data are presented with median with SD. *P* values < 0.05 were considered statistically significant. *p < 0.05, **p < 0.01 (mixed-sex vs. single-sex); ^#^*p* < 0.05, ^##^*p* < 0.01, ^###^*p* < 0.001 (naive vs. infected).

Female priming shows a significant upregulation of Th1-associated genes *tnf-α*, *il-1β* and *ifn-γ* compared with the mixed-sex infected control group. Male pre-infection induced a significant upregulation of *tnf-α* and *ifn-γ* expression. Male priming further leads to a significantly higher *ifn-γ* expression compared to -/mf control group, whereas relative expression of *tnf-α* in the non-primed group (-/mf) is nearly the same after single-sex priming. The relative expression of Th2-associated genes exhibits considerable interindividual variation and no consistent pattern, nevertheless, there was a significant increase in *il-33* expression after male-only and female-only infection compared to the bisexual control group. Genes associated with regulatory T cell response (*foxp3*) and the shift to it (*il-10*) are significantly increased in the male primed group. Expression of repair and resolution associated genes *hmox-1* and *retnla* was significantly upregulated in single-sex primed groups, whereas priming with female cercariae resulted in a greater increase of *retnla* expression (Fig 3B). *Icam-1* and *nos2* were significantly higher expressed in the single-sex primed groups, compared to the double bisexual infected mice. Furthermore, male priming leads to a significant decrease in the inflammatory *icam-1* expression compared to the non-primed group. Inflammatory and activation associated gene expression of *icam-1* and *nos2* is the highest in the non-primed control group. The lowest expression of all analyzed genes, except for *il-13*, was observed in the double bisexual infected group.

### Differences within the immune landscape between the individual infection groups are visible in immune cell and T cell compositions and densities

To investigate the cell composition and the associated immune environment of *S. mansoni* primed murine lungs during bisexual challenge infection, we performed flow cytometric analyses focusing on T memory cell subsets. Fig 4A shows tSNE visualizations of all investigated groups with all leukocytes combined.

**Fig 4:**
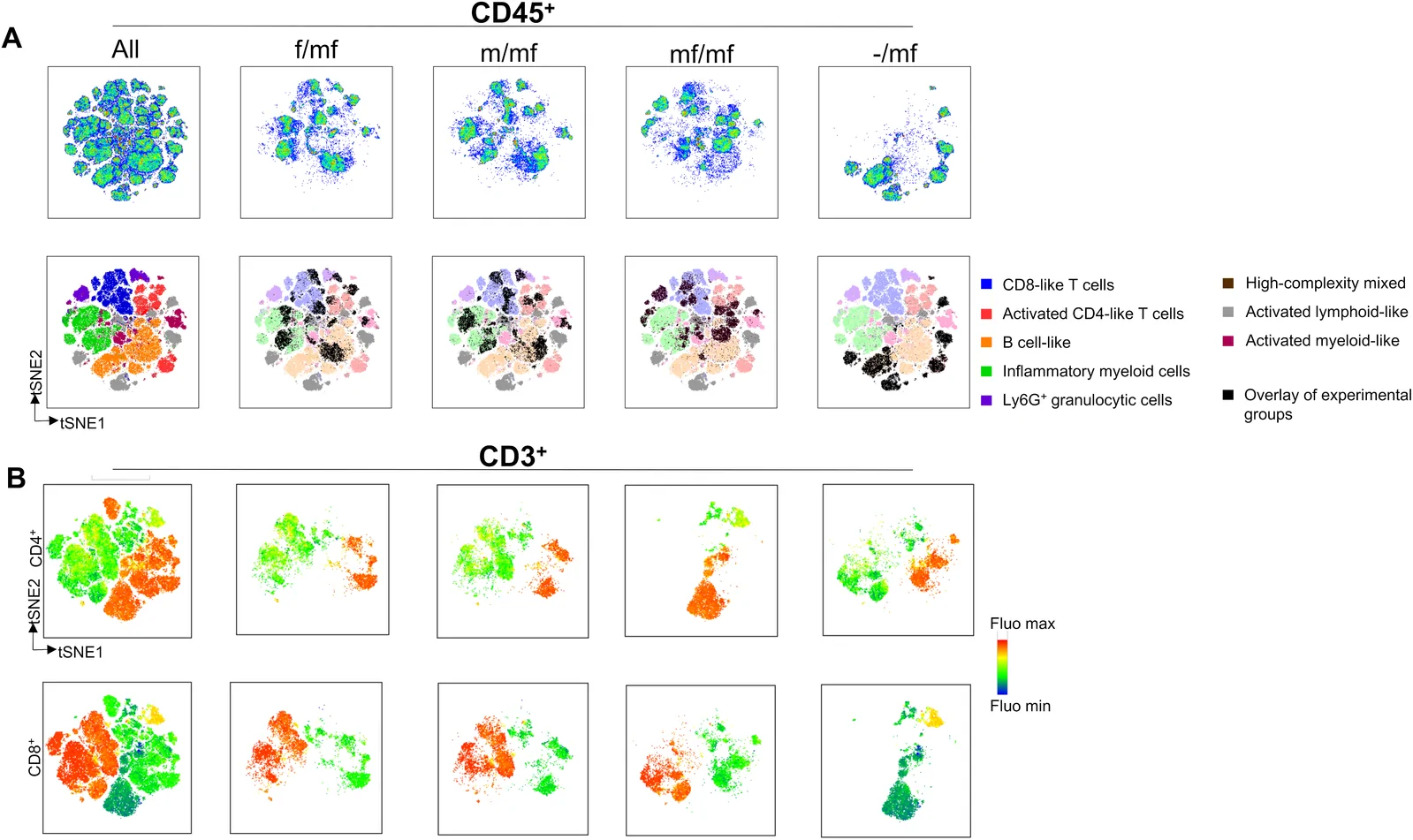
tSNE and FlowSOM analyses reveal distinct pulmonary immune architectures following different priming conditions. (**A**) tSNE visualization of concatenated pulmonary CD45^+^ live immune cells from all experimental groups followed by FlowSOM clustering analysis. Upper panels show density distribution of immune cells within each experimental group, whereas lower panels display annotated cluster populations including CD8-like T cells, activated CD4-like T cells, B cell-like populations, inflammatory myeloid cells, Ly6G⁺ granulocytic cells, activated lymphoid-like and activated myeloid-like clusters. Black overlay indicated the distribution of the respective experimental group within the overall cluster landscape. (**B**) tSNE visualization of concatenated pulmonary CD3 ⁺ T cells displaying CD4 and CD8 fluorescence intensity across experimental groups. M: male; f: female; mf: male and female; -: no infection. Color scale indicates relative fluorescence intensity from low (blue/green) to high (orange/red) expression levels. Equal numbers of events per sample were concatenated prior to tSNE analysis.

There are clear differences in cell density within the different populations visible between the primed groups and the non-primed control group, as well as between the bisexual and single-sex primed groups. Within this illustration eight populations could be identified, which refer to CD8-like T cells, activated CD4-like T cells, B cell-like, inflammatory myeloid cells, Ly6G^+^ granulocytic cells, high-complexity mixed, activated lymphoid-like cells and activated myeloid-like cells, respectively. For better visualization all groups were separately overlayed (black color) onto the cluster map in black to highlight the differences in cell populations between the individual infection groups. Especially between the non-primed and the primed groups, there were differences within the cluster referred to Ly6G^+^ granulocytic cells, activated lymphoid-like cells and the distribution within the B cell-like cluster. Furthermore, the density of the cluster referred to as inflammatory myeloid-like cells differ between single-sex primed groups, as well as in the clusters of activated CD4-like T cells and CD8-like T cells. To determine the T cell subpopulations in more detail, tSNE plots of all CD3^+^ cells are depicted for all groups analyzed (Fig 4B). Furthermore, the fluorescence intensity signal of CD4 and CD8 was overlaid to illustrate the differences between the distribution of CD3^+^ cells within the individual groups. It was observed that male and female priming lead to a higher density of CD8^+^ cell populations and lower CD4^+^ cell density compared to the bisexual control group. These findings could be confirmed by significantly lower CD4^+^ cell frequencies in the male primed group and lower frequencies in female primed mice, as well as significantly higher CD8^+^ cell frequencies in single-sex primed groups compared to the bisexual control (Fig 5B).

**Fig 5:**
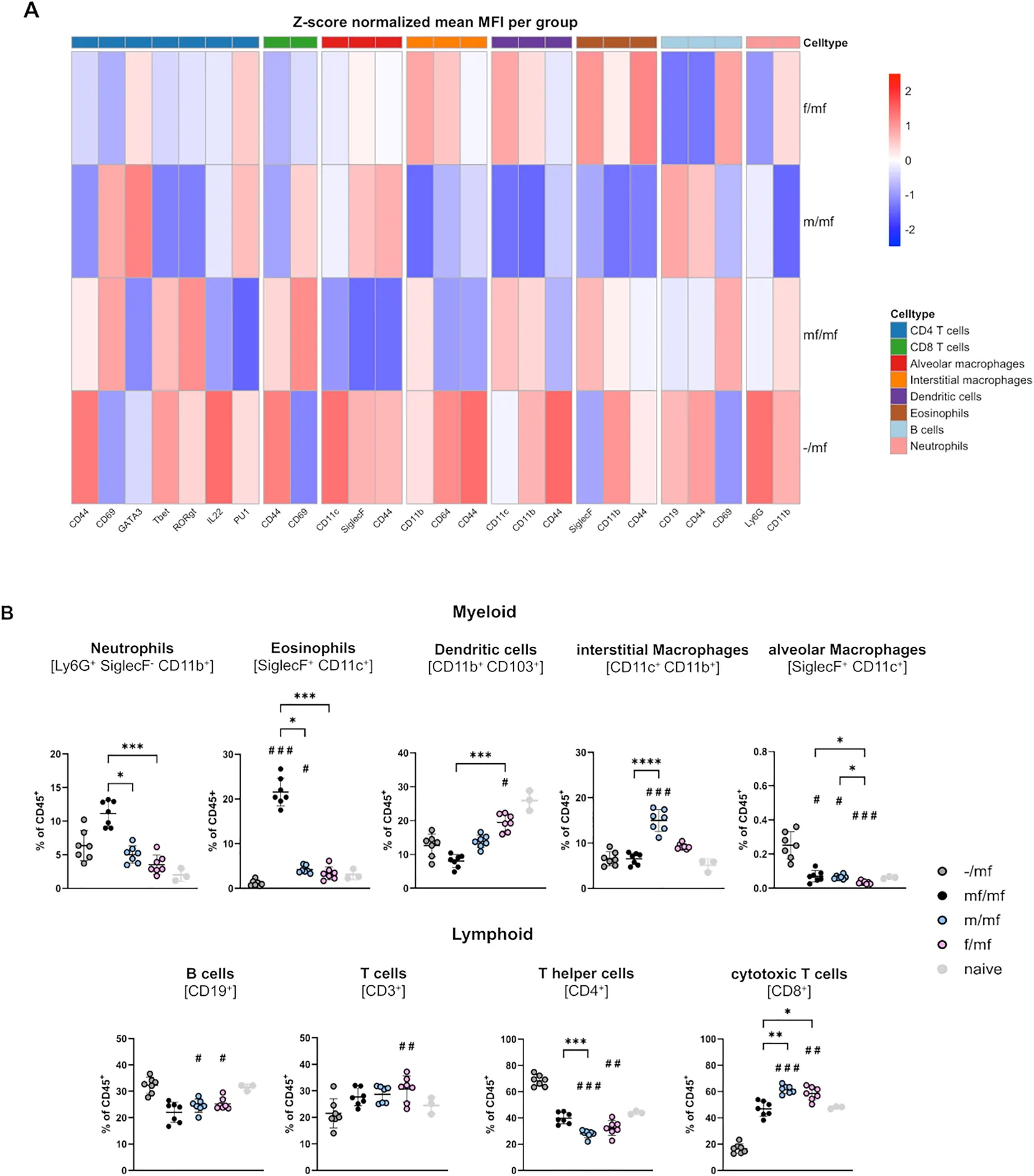
Single-sex and bisexual priming differently shape pulmonary immune activation patterns and immune cell composition during challenge infection. **(A)** Heatmap showing z-score normalized mean fluorescence intensity (MFI) of selected activation and lineage-associated markers across pulmonary immune cell populations isolated from experimental groups following challenge infection. Marker expression patterns are displayed for CD4^+^ T cells, CD8^+^ T cells, alveolar and interstitial macrophages, dendritic cells, eosinophils, B cells and neutrophils. Red indicates increased and blue decreased relative marker expression. **(B)** Quantification of pulmonary myeloid and lymphoid immune cell populations among CD45^+^ cells following challenge infection. Myeloid populations included neutrophils (Ly6G^+^SiglecF^-^CD11b^+^), eosinophils (SiglecF^+^CD11c^+^), dendritic cells (CD11b^+^CD103^+^), interstitial macrophages (CD11c^+^CD11b^+^) and alveolar macrophages (SiglecF^+^CD11c^+^). Lymphoid populations included B cells (CD19^+^), T cells (CD3^+^), T helper cells (CD4^+^) and cytotoxic T cells (CD8^+^). M: male; f: female; mf: male and female; -:no infection. Data are shown as median with SD. P values < 0.05 were considered statistically significant. *p < 0.05, **p < 0.01, ***p < 0.001 (mixed-sex vs. single-sex); ^#^*p* < 0.05, ^##^*p* < 0.01, ^###^*p* < 0.001 (naive vs. infected).

### The immune response between the infection groups differs in their organization and activation status

The heatmap analysis shown in Fig 5A revealed distinct group-specific patterns of marker expression across immune cell populations. Importantly, these differences reflect relative changes in marker expression between groups rather than absolute activation states. The map includes CD4^+^ and CD8^+^ T cells, alveolar and interstitial macrophages, dendritic cells, eosinophils, B cells and neutrophils and combined them with markers as CD44, CD69, CD11b and markers for T helper cell subpopulations as GATA3, Tbet and IL22. The non-primed control group -/mf shows a broad upregulation of multiple markers associated to cell activation across multiple cell populations. The male primed group, m/mf, displayed a more pronounced expression of activation markers within the T cell population, particularly within CD4 and CD8 T cell-like clusters. In contrast, the female primed group, f/mf, exhibit a more distributed pattern of marker expression across both lymphoid and myeloid-associated clusters, suggesting a less T cell-focused, but more broadly organized response to the challenge infection. The -/mf group served as the primary reference for changes associated with prior infection, whereas the mf/mf group provided an additional comparison representing recall responses following bisexual priming. In Fig 5B immune cell frequencies indicate that immune cell compositions between single-sex primed groups differ clearly from mf/mf. Within the myeloid panel, shown in Fig 5B, the cell frequencies of neutrophils and alveolar macrophages were significantly lower in the single-sex primed groups compared to the bisexual primed control, whereas female primed mice showed a lower percentage of cells within these groups than the male primed group. Female pre-infection led to the highest percentage of dendritic cells compared to the other groups, as well as significantly higher frequencies compared to the bisexual control. However male pre-infection leads to a significantly higher percentage of interstitial macrophages compared to the mf/mf control. Therefore, despite similar gene expression profiles, immune responses of f/mf and m/mf were not identical. Although CD3^+^ T cell frequencies remain broadly similar, single-sex priming showed an increased CD8^+^ T cell abundance, whereas CD4^+^ T cell frequencies were lower compared to the mf/mf group. In combination with the heatmap in Fig 5A the results indicate that subsequent differences in CD4-associated responses are more driven by activation state rather than cell abundance, which is why T cell subsets were analyzed for deeper understanding of the composition of subgroups within the individual infections. Fig 6A shows subsets of CD4^+^ T helper cells.

**Fig 6:**
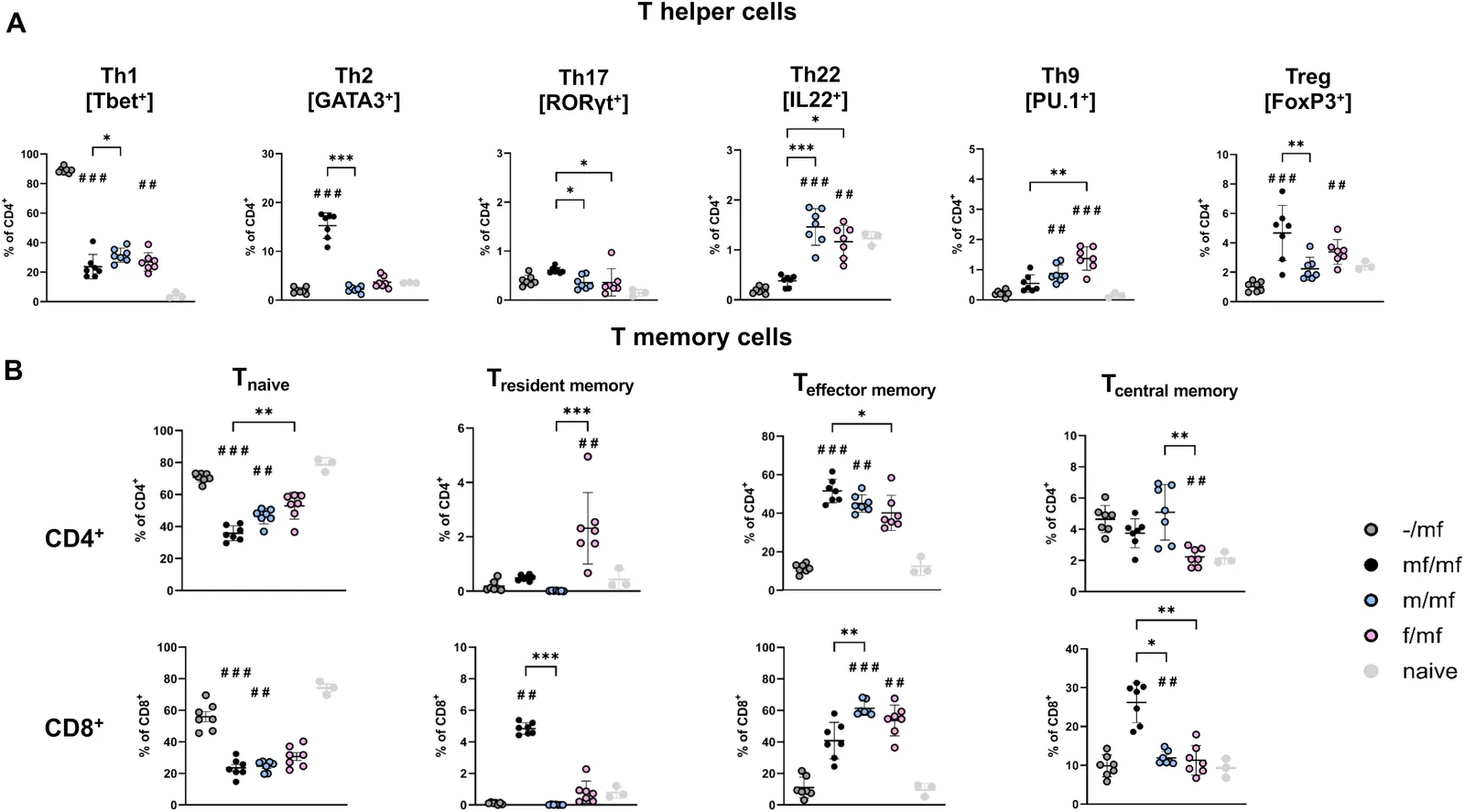
Unisexual and bisexual priming differently shape pulmonary T helper cell polarization and T cell memory architecture during challenge infection. **(A)** Quantification of pulmonary CD4^+^ T helper cell subsets following challenge infection. T helper subset populations were defined as Th1 (Tbet⁺), Th2 (GATA3⁺), Th17 (RORγt⁺), Th22 (IL22⁺), Th9 (PU.1⁺) and regulatory T cells (FoxP3⁺) among CD4 ⁺ T cells. **(B)** Quantification of pulmonary CD4⁺ and CD8 ⁺ T cell memory subsets following challenge infection. Analyzed cell populations were naive T cells, resident memory T cells, effector memory T cells and central memory T cells. Th: T helper; m: male; f: female; mf: male and female; -: no infection. Data are shown as median with SD. *P* values < 0.05 were considered statistically significant. *p < 0.05, **p < 0.01, ***p < 0.001 (mixed-sex vs. single-sex); ^##^*p* < 0.01, ^###^*p* < 0.001 (naive vs. infected).

Results indicate that male pre-infection leads to significantly higher frequencies of Th1 and Th22 cells compared to the bisexual control. Furthermore, the mf/mf control group showed the highest percentage of Th2 and regulatory T cells. Female priming lead to a slightly higher Treg cell abundance compared to the male primed group, however there was no statistical significance observed. Single-sex infection resulted in a significantly higher Th22 cell frequency compared to control groups mf/mf and -/mf, whereas male pre-infection leads to a higher abundance of cells than female priming. The results of Th9 cells show higher cell frequencies with single-sex priming, with female primed group exhibiting significantly higher frequencies than the mf/mf control. Single-sex priming led to a significant decrease in Th17 cell abundance compared to the mf/mf primed group. Fig 6B showed that single-sex primed groups displayed an increased frequency of naive CD4^+^ T cells compared to the mf/mf group. Not only was the frequency of naive CD4^+^ T cells significantly higher in the female primed group, but T resident memory cell abundance was also increased, whereas all other groups showed levels comparable to the -/mf control group. Furthermore, CD4^+^ T effector memory cell frequency was the highest within the mf/mf group and the lowest in the f/mf group, but all primed groups showed higher cell frequencies than the -/mf control group. CD4^+^ T effector memory cell frequencies in the -/mf group were comparable to those observed in naive mice. The abundance of T central memory cells was significantly elevated in the male primed group compared to the female primed group, which were nearly at the same level as in the naive mice. The CD8^+^ compartment indicates that priming in general leads to a decrease in the naive CD8^+^ cell pool. Furthermore, bisexual priming leads to the highest frequency of CD8^+^ resident memory T cells compared to the other groups. The frequency of T effector memory cells was significantly higher after male priming compared to bisexual priming and all primed groups showed higher cell frequencies than the -/mf control group. Bisexual priming led to a significantly higher T central memory cell frequency, but exhibited no differences between male and female priming which showed similar cell frequencies as the -/mf control group.

## Discussion

The aim of this study was to investigate how priming with single-sex or mixed-sex cercarial infections influences the immune response upon a bisexual challenge infection. We compared worm burden, key regulatory gene expression and changes in lung immune cell compositions during the pulmonary migration phase of the challenge larvae.

Our results demonstrate that prior infection markedly shapes the host response to reinfection in a sex-dependent manner. Male priming predominantly affected T cell composition and memory differentiation, whereas female priming was associated with broader changes across innate immune cell populations. The most pronounced differences between the two single-sex priming conditions became apparent within the pulmonary T cell compartment, particularly in the organization of memory T cell subsets.

In our previous study, we demonstrated that single-sex *Schistosoma mansoni* infection induces distinct pathological outcomes upon subsequent bisexual challenge infection [39,40]. Male priming was associated with significantly lower worm and egg burdens in the liver, whereas female priming resulted in attenuated Th2-mediated pathology, characterized by reduced liver fibrosis and smaller hepatic granulomas. This effect was linked to increased CTLA-4 expression and subsequent suppression of T cell activity [43].

In the present study, worm burden was assessed during the pulmonary migration phase, six days after challenge. Female primed animals exhibited the highest pulmonary worm burden, whereas both male primed and bisexually primed mice showed significantly lower worm counts (Fig 2).

The comparatively low pulmonary worm burden observed in the mf/mf group may reflect an immune environment already conditioned by chronic exposure to worm and egg antigens during primary bisexual infection.

Compared with the well-characterized immune responses to eggs and adult worms, relatively little is known about the host response to migrating lung-stage *Schistosoma mansoni* larvae. Houlder et al described a mixed Th1/Th2 response as a hallmark for early pulmonary infection with mixed-sex *S. mansoni* cercariae [44]. Consistent with this observation, our previous work showed that single-sex infections induce largely a non-polarized Th1/Th2 response during the early lung-stage, followed by progressive Th2 polarization at later stages of infection [38]. Similarly, unisexual priming in the present study was associated with a relatively non-polarized Th1/Th2-related gene expression profile in the lung, whereas bisexual priming induced a more pronounced Th2-associated response, likely driven by the persistent egg-derived immune stimulation.

The expression of *foxp3* and *il-10* was significantly increased following unisexual priming, particularly in male primed mice. This finding suggests the establishment of a more regulatory pulmonary environment compared to bisexual priming, which has previously been associated with limited Treg involvement [28,44]. Elevated *il-10* and *foxp3* expression has been linked to impaired resistance against challenge infection [28,45]. However, despite increased expression of these regulatory markers, male priming was associated with lower pulmonary worm burden than female priming. This apparent discrepancy indicates that additional immune mechanisms, beyond classical regulatory pathways, contribute to the altered outcome following reinfection.

Protective immunity induced by radiation-attenuated (RA) cercariae has been associated with enhanced pulmonary inflammation and increased tissue expression of ICAM-1 and mRNA of iNOS during challenge infection [46]. In contrast, bisexual priming in our study was characterized by significantly lower pulmonary expression levels of *icam-1* and *nos2*, suggesting a comparatively restrained inflammatory response. Previous work demonstrated that tissue damage and repair-associated genes are already upregulated as early as four days after single-sex infection [38]. Likewise, unisexual priming resulted in elevated expression of several tissue remodeling and repair-related genes compared with bisexual priming.

These observations suggest that single-sex infection is capable of priming pulmonary immune responses in a manner that shares certain features with RA vaccination, despite the antigenic differences between skin- and lung-stage schistosomula [47]. Appropriate regulation of pulmonary innate immunity is known to be critical for both host survival and successful parasite migration [48]. During lung migration of *Schistosoma japonicum*, activation of innate immune pathways together with wound healing responses involving *hmox-1*, *retnla* and *ch25h* has already been described [49]. Although priming generally resulted in reduced *hmox-1* expression, single-sex primed animals maintained significantly higher *hmox-1* levels than bisexually primed controls and showed elevated *retnla* and *il-33* expression. As an epithelial alarmin released upon tissue stress, IL-33 contributed to tissue repair and the triggering of type 2 immune responses, including during schistosomiasis [50,51]. Together, these findings indicate that single-sex priming promotes a pulmonary immune milieu characterized by enhanced immune regulation and tissue remodeling rather than a strongly polarized Th2 response. Such alterations may influence the immunological landscape encountered by migrating schistosomula upon challenge infection.

Previous studies have demonstrated that both specific humoral and cell-mediated immune responses are essential for resistance against challenge infections [52,53]. Consistent with these findings, our tSNE analyses revealed marked differences in immune cell cluster composition between primed and non-primed animals, particularly within activated lymphoid-like and B cell-like populations (Fig 4A). Corresponding MFI heatmap values further highlighted distinct pulmonary marker expression profiles, with the non-primed animals displaying comparatively high expression of several activation-associated markers during the first antigen encounter, represented by the -/mf group (Fig 5A).

Unlike the single-sex primed groups, which represent recall responses in the absence of prior egg exposure, the mf/mf group reflects recall responses within tissue conditioned by chronic exposure to both worm and egg antigens. Consequently, the mf/mf group represents a different immunological state rather than an intermediate condition.

Male priming was associated with distinct marker expression patterns in T cells and alveolar macrophages. Alveolar macrophages are known to play an important role in resistance to challenge infections of vaccinated mice due to T cell and macrophage recruitment [54]. Although male priming predominantly affected the pulmonary T cell compartment, an increased proportion of interstitial macrophages indicated that selected myeloid populations were likewise influenced (Fig 5B).

In contrast, female priming mainly affected the activation characteristics of dendritic cells, eosinophils and interstitial macrophages rather than altering their frequencies, suggesting preferential remodeling of pulmonary myeloid cell populations (Fig 5).

The importance of T cell-mediated immunity in protection against schistosome challenge infections is well established, particularly the coordinated interaction between Th1, Th2, Th17 and regulatory T cell populations [55,56]. Among these, Th1 responses are considered critical for the elimination of migrating schistosomula through activation of macrophages and support of B cell-mediated production of complement-fixing and opsonizing antibodies that could result in host resistance [57–62]. In agreement with this concept, tSNE visualization revealed substantial differences in both the abundance and distribution of CD4^+^ and CD8^+^ cells between primed and non-primed animals (Fig 4B). Analysis of immune cell frequencies revealed that the overall proportions of CD3^+^ T cell remained largely comparable across the priming groups, while marked differences were observed in the relative representation of CD4^+^ and CD8^+^ T cells (Fig 5B).

Notably, male primed mice exhibited lower CD4^+^ T helper cell frequencies than bisexually primed mice, whereas both single-sex primed groups displayed increased CD8^+^ cytotoxic T cell frequencies compared to the bisexually primed group. Male priming was further associated with a higher frequency of Th1 cells and a lower frequency of regulatory T cells compared with bisexual priming upon challenge infection (Fig 6A). This observation is particularly interesting given that schistosomes are known to promote Treg expansion and IL-10 production as mechanisms to suppress potentially protective Th1 responses [63,64]. Repeated male-only infections have been shown to induce expansion of CTLA4^+^ Tregs [65,66], a phenomenon associated with enhanced immune regulation and parasite persistence. In contrast our data suggest that single male priming infections may shift the balance toward a more protective immune profile, whereas repeated male-only exposure may promote regulatory mechanisms that favor parasite survival [66].

Further support for the importance of prior antigen exposure comes from studies by Coulson and Wilson, who demonstrated in radiation-attenuated cercarial vaccination models that pulmonary CD44high effector/memory T cells mediate rapid recall responses against challenge parasites [67]. Effector memory T cells rapidly migrate to sites of inflammation and provide immediate effector functions upon secondary antigen exposure. In contrast, central memory T cells possess little immediate effector function but serve as a proliferative reservoir that rapidly expands and differentiate into effector cells following secondary antigen encounter [68]. T_EM_ and T_CM_ are circulating cells, whereas resident memory T cells remain permanently within peripheral barrier tissues as the lung. They provide immediate local immune surveillance and initiate rapid protective responses without requiring recruitment from the circulation [69].

Our results show that especially male priming led to higher frequencies of CD4^+^ effector memory and central memory T cells compared with female priming. Within the CD8^+^ compartment, effector memory frequencies were similarly high following single-sex priming and exceeded those observed after bisexual priming, whereas central memory frequencies were highest in the bisexually primed group (Fig 6B). Given that protective immunity against *S. mansoni* has been attributed mainly to CD4^+^ T cell-mediated immune mechanisms, whereas depletion of CD8^+^ T cells has little effect on vaccine-induced protection, these differences within the CD4^+^ memory compartment may be of particular functional relevance [70]. The association between reduced worm burden following male priming and the enrichment of circulating CD4^+^ memory subsets suggests that it is the quality and organization of the pulmonary memory response, rather than its mere magnitude, that may contribute to protection against reinfection. In contrast, first infection with female *S. mansoni* resulted in a less intense CD4^+^ effector/central memory profile but a strong enrichment of CD4^+^ resident memory T cells.

Interestingly, resistant human individuals show higher frequencies of naive CD4^+^ and CD8^+^ cells and higher CD4^+^CD45RO^+^ memory cells. Furthermore, resistant individuals display higher CD4^+^ T central memory cells and lower CD4^+^ T effector memory cells compared to the susceptible control group [71]. Some, but not all, of these characteristics were also observed following male priming. In particular, the increased CD4^+^ central memory frequency resembles the profile described in resistant individuals, whereas the elevated CD4^+^ effector memory frequency differs from human pattern. These partial similarities support the possibility that male priming establishes memory characteristics associated with altered resistance, although the functional contribution of these subsets remains to be determined. Together, these observations support the concept that the sex of primary infection influences the organization of pulmonary recall immunity.

While the observed differences in pulmonary immune organization indicate distinct recall responses depending on the priming condition, the interpretation of pulmonary parasite burden requires caution. The lung represents a highly dynamic transit compartment during schistosome migration, with larvae remaining detectable between approximately days 4 and 20 post infection [27]. In the present study, pulmonary worm burden was assessed at a single time point, corresponding to the previously determined peak of lung migration upon mixed-sex infection. Consequently, the measured parasite burden represents only a transient snapshot rather than a definitive infection outcome, such as adult worm recovery from the portal system.

Differences in pulmonary worm burden may therefore reflect not only altered parasite elimination but also changes in migration kinetics, retention time within the lung or accelerated tissue passage following prior antigen exposure. Previous studies have already demonstrated that schistosome migration dynamics can vary according to both host background and parasite strain, further supporting the notion that pulmonary parasite burden does not necessarily predict final infection establishment [27,72,73]. Nevertheless, the overall pattern observed in this study is consistent with the hypothesis that male priming alters the pulmonary immune environment in a manner that may facilitate more efficient control of migrating schistosomula.

In summary, migration kinetics following single-sex infection are currently unknown and should be addressed in future longitudinal studies examining multiple time points during parasite transition. Functional validation of the identified memory subsets, including assessment of cytokine secretion and recall capacity, will be necessary to determine their precise contribution to protection. The present study provides a detailed characterization of pulmonary immune responses following single-sex priming and establishes a foundation for further investigation of the underlying mechanisms. As our analyses were restricted to the pulmonary compartment, the present study does not distinguish locally established immune memory from systemic immune changes originating in secondary lymphoid organs. Future studies extending the analysis to draining lymph nodes and secondary lymphoid organs may provide additional insights into systemic recall responses following single-sex priming. Furthermore, a more detailed investigation of host-parasite interactions during lung migration, including potential sex-specific differences between male and female larvae, may further elucidate the mechanisms underlying priming-associated immune modulation and protection.

## Conclusion

Our findings demonstrate that male and female single-sex *Schistosoma mansoni* priming establishes distinct pulmonary immune recall profiles during subsequent bisexual challenge infection. Male priming was associated with reduced pulmonary worm burden and an enrichment of circulating CD4^+^ effector and central memory T cells, whereas female priming promoted CD4^+^ resident memory T cells together with broader alterations in pulmonary myeloid populations. These findings suggest that the organization of pulmonary CD4^+^ T cell memory may contribute to the distinct pulmonary worm burden observed following male or female single-sex priming.

## Supporting information

S1 Fig Gating strategy of lymphoid and myeloid cells. Eosino: Eosinophils; AM: alveolar Macrophages; MO: Macrophages; IM: interstitial Macrophages; cDC: conventional Dendritic Cells; Th cells: T helper cells; T cyt: cytotoxic T cells; T_EM_: T effector memory cells; T_CM_: T central memory cells; T_naiv_: naive T cells; T_RM_: T resident memory cells; T_reg_: regulatory T cells.
(DOCX)

S2 Fig Heatmap used for cluster assignment in the tSNE plot generated by FlowJo’s Cluster Explorer following FlowSOM analysis of flow cytometry data. Cluster 6,11 = CD8-like T cells; Cluster 2, 10, 7 = activated CD4-like T cells; Cluster 12, 18 = B-cell like; Cluster 8, 13 = inflammatory myeloid cells; Cluster 14,15 = Ly6G^+^ granulocytic cells; Cluster 1, 3, 4, 9 = activated lymphoid-like; Cluster 5, 16 = activated myeloid-like; Cluster 17 = high-complexity mixed.
(DOCX)
